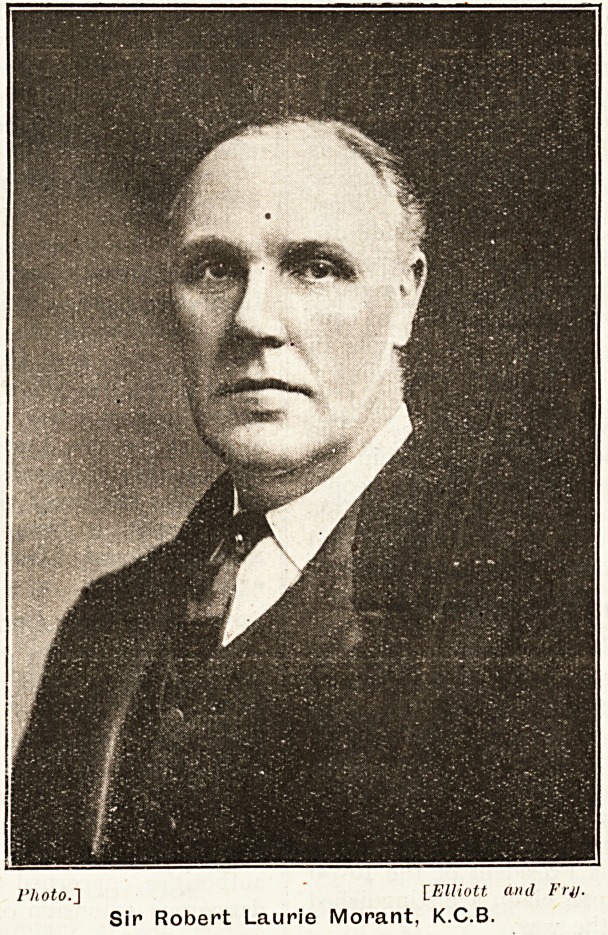# Hospital and Institutional News

**Published:** 1920-03-20

**Authors:** 


### HOSPITAL AND INSTITUTIONAL NEWS.

### DEATH OF SIR ROBERT MORANT.

THE death from pneumonia in his fifty-seventh year of Robert Laurie Morant, Chief Permanent Secretary to the Minister of Health, removes one of the outstanding personalities of his day, and a public servant whom the nation and the newly-formed Ministry of Health can ill afford to lose. An exceptional man in many ways, Morant was very exceptional indeed in attaining the highest ranks of three separate Government Departments without having been originally a "covenanted" member of the Civil Service at all. He was, in fact, thirty-two years of age before he first got his foot on the ladder in the Education Office; the interval between his Oxford days (he took a theology course there, strange to say) had been filled in educational work, first in England and later in Siam, where he made so great an impression that he was dubbed the uncrowned King of Siam. Having got his opportunity at the Education Office, he soon pushed himself to the front by that combination of personality, restless energy, and intellect which made him remarkable in any company. Of course, he made enemies: no man of his type could avoid doing so. He drove his subordinates relentlessly, though not nearly so relentlessly as he drove himself; and the writer of the *Times* notice is probably quite right in attributing his comparatively early death partly to persistent overwork and exhaustion.

*Photo.*] [*Elliott and Fry.*

**Sir Robert Laurie Morant, K.C.B.**

### MEDICAL AND INSURANCE PROBLEMS.

THEN came yet another field of work. Mr. Lloyd George took him from the Education Office, where in only eight years he had risen to be Permanent Secretary, to be Chairman of the National Health Insurance Commission. Here Morant had many medical problems to deal with; he tackled them with conspicuous success, because, although he had no medical training, he was a man with a gift for grasping principles and for adhering to them unswervingly. More and more he became wrapped up in the great principle of co-ordinating the various public health activities of the nation under a single Ministry; and he lived not only to see the Ministry of Health established, but also to become its first Chief Secretary. The hospital crisis, it is stated, was receiving his closest attention at the time that he fell ill; how he would have proposed to solve it will never now be known. His great gifts were in many ways attuned sympathetically to the voluntary hospital system, in that he was a hater of "eyewash" and of officialism, and a keen lover of real genuine efficiency. With all his breadth of vision, he was also exacting over minute details; nothing was too small to be worth taking trouble over. To replace this outstanding man at the Ministry of Health is certain to be difficult; his successor, whoever he may be, has a difficult standard to live up to.

### NATIONAL CHAIN OF HOSPITALS.

SIR NAPIER BURNETT, the director of the hospital services under the control of the Red Cross, has given a representative of the Press an outline of the scheme which is being organised to relieve provincial hospitals which are victims of the effects of what is known as the "hospital crisis." At present the Red Cross organisation is concentrating upon (1) a survey of the position; (2) a widespread appeal for financial assistance; (3) co-operative buying on analysis; and (4) a reconstructed scheme of hospitals by setting up co-ordinated groups of institutions. Sir Napier and his assistants are already getting an idea of the general position, and from information already to hand, there is apparently overlapping in some respects. As one instance of this, it is stated that in a certain district one hospital had an enormous waiting-list, while others had vacant beds. Again, loss resulted by hospitals competing with each other in buying supplies and in outbidding each other in the matter of the salaries of hospital workers. The Red Cross hope before long to be in a position to buy in bulk at the ship's side at cheap rates, and then distribute the goods throughout the country. Co-operative buying has been tried in London on a small scale. It is not fair to say that it failed, but, at any rate, the system was discontinued. The fault probably lay in the fact that there was co-operative buying without co-operative distribution, and in this important way it differed from the present Red Cross scheme.

### THE PROVINCES TO BE HELPED FIRST.

It is only fair that the potential benefactor of hospitals should understand clearly that the Red Cross Scheme of help applies at present only to provincial hospitals. In London there is the King's Fund, which from its own resources helps the London hospitals in the matter of maintenance, and from special Red Cross grants is about to help them in the matter of extraordinary expenditure on building and improvements. It is as well not to make too much of this point, as King Edward's Fund has not an elastic income, and can but give approximately what it gave before the war, although the requirements of the recipients are doubled. It is fortunate in a sense that the money for London from Red Cross funds will have to be conserved, as any estimates for building at the present time are so terrifying in expensiveness that it is only the boldest and richest that can embark on such expenditure. Alterations should, possibly, be in the direction of revenue-producing ventures, such as the preparation of wards with cubicles for the reception of contributing patients. Sir Napier Burnett is quite in favour of such a move, in addition to the ordinary patient of a lower financial grade being asked to contribute towards the cost of maintenance. The "new poor," in his opinion, now come within the category of voluntary hospital patients, as people with fixed moderate incomes are no longer able to pay the expenses of private nursing homes, and in a proper system there must be a place for them.

### SIR GEORGE MAKINS'S APPOINTMENT.

We read that Sir George Makins has been appointed to succeed the late Sir Wm. Osler as Chairman of the Fellowship of Medicine. The history of the Fellowship and its already achieved success is well known to all. Sir Wm. Osler played a devoted part in bringing about this eminently satisfactory beginning, and even during his last illness had its future welfare prominently in mind. His successor is President of the Royal College of Surgeons, and can claim a distinguished record of service during the war.

### CHADWICK PUBLIC LECTURES.

Following on the presentation of the Chadwick Naval and Military prizes of £100 each and a gold medal to both Surgeon-Commander E. L. Atkinson, D.S.O., R.N., and Brigadier-General W. W. O. Beveridge, C.B., C.B.E., D.S.O., A.M.S., in the Lecture Hall of the Royal Society of Arts, on Monday, March 8, the first of a series of three Chadwick Lectures was given by Lieutenant-General Sir John Goodwin, K.C.B., C.M.G., D.S.O., F.R.C.S., Director-General of the Army Medical Service; Sir William Collins, K.C.V.O., M.D., M.S., Chairman of the Chadwick Trustees, being in the Chair. The lecturer dealt with the history of hygiene, more especially Army hygiene, from the earliest days up to the time immediately preceding the recent war. The ravages wrought by disease and sickness during various campaigns of the eighteenth and nineteenth centuries, and their effects upon armies in the field, were detailed in succession, and the various steps in improvement, more especially as regards education and organisation, were explained. Emphasis was laid on the lessons in disease prevention which were learned during the South African War, and the measures which were taken to improve matters between that campaign and the recent European War. The next lecture of the series, on March 15, will deal with Army hygiene during the recent war, the developments attained, and the lessons learned, while the third and last lecture, on March 22, will deal with the future of Army hygiene and its relation to that of the civil community. Further information about this and other Chadwick Lectures may be obtained of the Secretary at the offices of the Trust, 40 (6) Queen Anne's Chambers, Westminster.

### IMPROVEMENT IN MATERNITY CASES.

The Ministry of Health has sanctioned a proposal of the Southwark Board of Guardians to remove all their maternity cases from Newington Institution to their infirmary, where a special ward will be set apart for them. This is subject to a proviso that the Guardians shall retain at the Newington Institution a bed and maternity nurse for emergency cases. The alteration will remove, as far as possible, these cases from the workhouses. The number of Boards of Guardians in the Metropolis who still treat their maternity cases in their workhouses is being gradually reduced. Bermondsey Board of Guardians some time ago adopted a similar policy.

### SMALL-POX REAPPEARS.

News comes of a definite outbreak of small-pox on both north and south sides of the Thames Estuary, the names of Poplar and West Ham being specifically mentioned. At the time of writing, neither the number of cases, their distribution nor circumstances give any obvious cause for alarm among the general public. We have every confidence that, as in previous outbreaks, the local authorities concerned can be relied upon to limit dangerous extension of the disease.

### A WORD ON THE NOTTINGHAM APPEAL.

So long as the lay press continues to create the panic which it professes to record in regard to the financial difficulties of the voluntary hospitals, it is difficult for us to find the space to describe every voluntary effort now being made to meet them. Of Cardiff, of Leeds, of Birmingham, we have written at more generous length; but among the active centres to which we have yet given a glance only is Nottingham, where an effort is being made to raise a further £20,000 a year. In a carefully drafted and delightfully illustrated appeal, the Nottingham General Hospital is laying its case before the public. A remarkable instance of success was recorded last week, when we mentioned that on a generous gift being made by a body of employees, the head of the firm *multiplied its annual subscription by ten.* We wish to hear more of the organisation of subscriptions from employers, and again recommend the Leeds scheme to Mr. P. M. MacColl and his committee. We have one criticism to offer on an otherwise excellent appeal. Its readers are urged to support the voluntary principle because a voluntary hospital "is charged with some indefinable element difficult to describe." That is both weak and inaccurate. The element which inspires a voluntary hospital is not indefinable, nor is it difficult to describe. It is the element of personal service, which puts the patient first and self second; which desires to give and not to gain; whose ambition is to help, not to be paid or promoted. The atmosphere of a voluntary hospital is the atmosphere of an institution from which the commercial code of morality and standard of conduct have been absolutely exorcised. If the hospitals cannot define their own cherished possession, they will hardly defend it successfully. It is essential that they should know and be able to describe their own character.

### FINANCIAL PROGRESS IN LIVERPOOL.

Speaking at a meeting of the David Lewis Northern Hospital, Liverpool, Mr. T. Nowell, honorary secretary of the Hospital Saturday Fund, stated that the fund expected to distribute at least £10,000 *more than last year* to the hospitals. The weekly collections in the workshops, and only about half the financial year had run, showed already an increase of nearly £1,000. In addition a donation of £5,000 had been received for the Joint Saturday and Sunday Fund, beside another gift of £1,000. Mr. Burton W. Ellis, the Lord Mayor of Liverpool, from whose fund much is hoped, said at the same meeting that local authorities like the Corporation, the Select Vestry, and the Board of Guardians were considering with sympathy a proposal to make grants to the hospitals. Since the Corporation made grants for building, he did not see why it should not also make grants towards hospital maintenance. The voluntary system must be maintained, and it would be a great mistake to subvert it.

### MR. WADE DEACON ON THE OUTLOOK.

One advantage of the voluntary system is the decentralisation which it involves, whereby sometimes London gives a lead to the provincial hospitals, and as often as not the provinces give a lead to London. At the moment, perhaps, the provinces are more forward in the matter. The Liverpool Royal Infirmary, which would have been in serious financial straits but for the receipt of an anonymous gift of £20,000, still owes nearly £5,000 to the bank. The deficit on the year's work was nearly £12,000, a figure which is all the more alarming when we remember that the ordinary receipts, largely thanks to the grants made to Service patients, were increased by £1,500 on the previous year. It needs, in fact, an increase of £45,000 a year. The Lord Mayor's fund, which has raised £46,000, should make a grant to the Royal Infirmary sufficient at least to clear the debt; but maintenance is the chief problem. Mr. Wade Deacon, however, is not discouraged. He hopes that the re-direction of Red Cross organisation towards the maintenance of hospitals in peace will do much. Can he give us any news of local effort in Liverpool? The impulse should come not only from London, but from each centre. Who is devoting his energies to this in Liverpool? Mr. Wade Deacon also advocates a system of co-ordination between different institutions, whereby special and post-operative treatment may be continued in institutions other than those which the patient first entered. It would be interesing to know how far Mr. Deacon has a definite programme on this point.

### THE JACKSON BABY.

As we go to press we learn that public opinion has been too strong for the Bramley Board of Guardians in their action which we commented on at length last week (page 554). Over 1,000 letters of protest were received by the Clerk to the Board, and in the result, after a lengthy interview with the General Secretary of the Yorkshire District Poor-law Workers' Union, Mr. G. Vincent Evans, the Board has rescinded its resolution by which Mr. and Mrs. Jackson, the porter and porteress of Bramley Union, Yorkshire, were to be deprived of their child, a baby of a few months old. We congratulate the Yorkshire District Poor-law Workers' Union, the public at large, and Mr. and Mrs. Jackson on the result of their protest, and we hope the lesson will be taken to heart by other Boards of Guardians throughout the country.

### THE WILL AND THE WAY AT BARNSLEY.

The Beckett Hospital, Barnsley, has had an encouraging degree of success in its efforts to meet its financial difficulties. The fund of the Lord Mayor, Colonel Roley, which was started to raise £20,000 to relieve the hospital of debt, has already received £13,000 in gifts and promises. To improve the maintenance, new contributions from the miners have been organised, and already £3,000 has been obtained from new contributions. This and other inspiriting examples reach us week by week, which show that the voluntary system retains its old life and vigour when men who really believe in it, like Mr. A. Whitham, are at the helm. The real need, in fact, is to find the men. The "problem" is the amusement of the pessimists, who create the scare which they profess to chronicle.

### EMPLOYERS' SUBSCRIPTIONS AT KEIGHLEY.

We hope that a beginning has been made to organise an employers' subscription fund at Keighley for the benefit of the Victoria Hospital there. The idea has been mentioned by Mr. J. E. Haggas, the honorary treasurer; but will he follow it? A model scheme, fully described to its smallest details in The Hospital of February 7, p. 437, has been successfully started at Leeds, and we make no apology for once again drawing attention to it. We suggest, in fact, that there is more to be hoped from such a scheme than from Mr. Haggas's suggestion, useful as it was, that if the local employers would subsidise their men's contributions by a subscription equivalent to one-third much would be done for the hospital. This is well, as far as it goes; but the merit of the Leeds scheme was the interest which it gave to the employers, the encouragement of a corporate feeling among them, and the simplicity of its working. Now these invaluable qualities were the reward of careful thought, of attention to detail, of thoroughness. We invite Mr. Haggas to study the scheme, because we believe that he will rise from his application to master it a less anxious and more cheerful man than his difficulties as treasurer allow him to be at present.

### A MENTAL HOSPITAL REFORM.

Dr. H. de M. Alexander, of Kingseat Mental Hospital, lately interested a lay audience in the reform, past and prospective, of the mental hospital. Speaking at the Rotary Club, Aberdeen, he urged the importance of encouraging voluntary patients, in order to secure more early treatment than the present state of the law allowed. As the Medico-Psychological Association had suggested, the rate-assisted mental case should be removed from the Poor Law, and such a patient should have the right to enter a mental hospital voluntarily. No reform would do more to encourage early treatment. Such an address should help to remove the prejudice by dispelling the ignorance of most laymen in respect of asylums. We hope that addresses of a similar kind may be made to lay audiences elsewhere. Our experience is that any audience enjoys an address by a speaker of repute as much as an evening at the theatre. The popularity of the lecture is much underrated.

### PATIENTS AND BOARD MONEY.

The proposal that patients should be invited to pay a weekly sum towards the cost of their food when in hospital (for 10s. a week would not cover a sick person's careful diet) is an interesting application of a recent principle. One of the concessions granted of late years to institutional staffs has been the present of board money to them when away on holiday. Such a concession would have made our grandfathers stare, for to them a holiday itself was a very great concession. Even now in commerce many a man receives nothing when he is away ill. We have regarded this concession of board money as a surreptitious bonus in lieu of proper wages, for wages can hardly be regarded as reasonable which make a holiday an almost impossible expense. The precedent thus created, which will probably extend to commercial life in time, is now being applied to patients, who are to be charged a sum more or less equivalent to that which they would pay were they at home. The deciding question, therefore, seems to be, How many hospital patients are receiving their wages or benefit equivalent thereto when on the sick-list?

### OVER-ZEALOUS APPEAL HELPERS.

A hospital or charity which accepts the services of helpers not directly upon the appeal staff is none the less responsible in a general way for the methods adopted by such friends and, to keep some control on over-zealous methods, should be cognisant of the character of any proposed campaign. One scheme, which we are sure could not meet with the approval of the great hospital it is intended to benefit, is to send on approval tickets for a forthcoming concert, leaving the recipient only two alternatives, one to buy the tickets and the other to pay the postage on returning them to the sender. This must cause irritation in many quarters and a tendency to lessen existing goodwill towards the hospital. It may be said that many of these charity concerts and functions cost far too much to get up. At the recent "Pan" ball held for St. Bartholomew's Hospital and the Reigate Homes for Soldiers' Babies, for one night's entertainment the organisers received a fee of £300, the orchestra was paid £178, and the rent and lighting of the Opera House came to £812. Out of takings amounting to a little over £5,000 only a sum of £1,823 found its way into the coffers of the charities concerned, a sum which was probably far less than the private expenses of the dancers on costumes, carriage hire, and the like. Apparently there was a terrible lot of expense and a terrible little of genuine charitable feeling.

### LORD BUTE'S GIFT TO ABERDARE.

Aberdare has to thank the Marquis of Bute for a munificent gift to the town. When the remaining portion of Lord Bute's Aberdare estate came lately under the hammer the auctioneer withdrew one lot, that comprising Abernant House (now the Aberdare General Hospital) and the adjoining ground of over three acres, as his lordship had decided to present it to the town. This decision was received with applause by the large company present at the sale, and Mr. Charles Kenshole, on behalf of the Hospital Committee and the townspeople, expressed their grateful thanks. This is another manifestation of the donor's benevolent attitude towards hospitals.

### THIS WEEK'S DRUG MARKET.

Price fluctuations have been rather numerous and, while several articles have moved downwards, the tone generally is firm, and so far as can be foreseen is likely to remain so, in view of the continued large demand for drugs for export. Ipecacuanha has reached a very high figure, and there is practically no relief of the scarcity. Lemon oil, bergamot oil, and orange oil have a strong upward tendency in prices. Nux vomica, thymol, phenazone, citric acid, and aloes are dearer. Menthol, Japanese refined camphor, and Japanese peppermint oil are still quiet, and prices are barely maintained. Cod-liver oil is slightly cheaper at the moment. Paraldehyde, benzoic acid, sodium benzoate, and barbitone are offered at rather lower rates.

## Figures and Tables

**Figure f1:**